# Supplementary material for: Patterns of Intron Gain and Loss in Fungi
Source: PLoS Biol. 2004 Nov 30;2(12):e422. doi: 10.1371/journal.pbio.0020422 (PMC532390; doi:10.1371/journal.pbio.0020422)
Supplement: Table S1 — Also available at http://genes.mit.edu/NielsenEtAl/. (4.3 MB ZIP). [file pbio.0020422.st001.zip › NielsenEtAl/html/994.html]

AN4915.1.NCU06410.1.MG06241.1.FG05501.1


```
 CLUSTAL W (1.82) Multiple Sequence Alignments - Introns Inserted


Sequence 1: NCU06410.1	236 aa
Sequence 2: FG05501.1	234 aa
Sequence 3: MG06241.1	238 aa
Sequence 4: AN4915.1	258 aa
Alignment Length: 258 aa
Number Identitical Residues: 173 aa
Alignment Score (without introns) 6721


MG06241.1 	MAD----GAAKPSSSVKLVLLGEAAVGK0SSLVLRFVNNDFQENKEPTIGA1AFLTQKCN
NCU06410.1	MAD-T--NAPKPSSSVKLVLLGEAAVGK0SSLVLRFVNNDFQENKEPTIGA1AFLTQKCN
FG05501.1 	MADST--NAPKPSSSVKLVLLGEAAVGK0SSLVLRFVNNDFQENKEPTIGA1AFLTQKCN
AN4915.1  	MSESTPANAPKPSSSVKLVLLGEAAVGK0SSLVLRFVNNDFQENKEPTIGA1AFLTQKCS
          	*::::.:.*.****************** ********************** *******.

MG06241.1 	LPARTIKFEIWDTAGQERFASLAPMYYRNAQAALVVYDLTKPTSLIKAKHWVAELQRQAS
NCU06410.1	LPTRTIKFEIWDTAGQERFASLAPMYYRNAQAALVVYDLTKPTSLIKAKHWVAELQRQAS
FG05501.1 	LPTRTIKFEIWDTAGQERFASLAPMYYRNAQAALVVYDLTKPTSLIKAKHWVAELQRQAS
AN4915.1  	LPSRTIKFEIWDTAGQERFASLAPMYYRNAQAALVVYDVTKPSSLTKAKHWVAELQRQAS
          	**:***********************************:***:** **************

MG06241.1 	PGIVIALVGNKLDLTSDGGDASGGGD-DDGDAEGAGDDS---------GDARKIPTEEAK
NCU06410.1	PGIVIALVGNKLDLTSD---SAGSAE-ASGGGDDNAEDS---------GDARKISTDEAK
FG05501.1 	PGIVIALVGNKLDLTGD---SSSVAG-ADG---EEGDDS---------GDARKVPTEEAQ
AN4915.1  	PGIVIALVGNKLDLTNDGGETPAETEREAGTEGDDADDNQEEQEDNTPGDARKVPTREAS
          	***************.*...:..      *     .:*........:.*****:.* **.

MG06241.1 	AYAEEEGLLFFETSAKTGFNVTEVFTAIANAIPETSLKSARGAGAGA------GAGRSGE
NCU06410.1	AYAEEEGLLFFETSAKTGHNVTEVFTAIANAIPETSLKSARGPGASH------AGVSRTE
FG05501.1 	AYAEEESLLFFETSAKSGHNVTEVFTAIANAIPETSLKSARGAGASN------AASRGSE
AN4915.1  	TYADEEGLLFFETSAKTGLNVTDVFTAIANAIPESSLKSGRGAGAGTGQTTLGGGGRSTD
          	:**:**.*********:* ***:***********:****.**.**. ..:: ...    :

MG06241.1 	QQSVSLNGPRDVEGKDGCAC
NCU06410.1	EQRVNLNGPRDLDAKEGCAC
FG05501.1 	EQRVNLGGPKDAGAKDSCAC
AN4915.1  	DSRVNLGDRGAATAKEGCAC
          	:. *.*..     .*:.***
```
